# The Bethe–Salpeter formalism with polarisable continuum embedding: reconciling linear-response and state-specific features†
†Electronic supplementary information (ESI) available: Cartesian coordinates of the compounds. See DOI: 10.1039/c8sc00529j


**DOI:** 10.1039/c8sc00529j

**Published:** 2018-04-05

**Authors:** Ivan Duchemin, Ciro A. Guido, Denis Jacquemin, Xavier Blase

**Affiliations:** a Univ. Grenobles Alpes , CEA, INAC-MEM, L_Sim , F-38000 Grenoble , France . Email: ivan.duchemin@cea.fr ; Email: xavier.blase@neel.cnrs.fr; b Laboratoire CEISAM – UMR CNR 6230 , Université de Nantes , 2 Rue de la Houssinière, BP 92208 , 44322 Nantes Cedex 3 , France; c Laboratoire MOLTECH – UMR CNRS 6200 , Université de Angers , 2 Bd Lavoisier , 49045 Angers Cedex , France; d Univ. Grenobles Alpes , CNRS , Institut Néel , F-38042 Grenoble , France

## Abstract

The Bethe–Salpeter equation (BSE) formalism has been recently shown to be a valuable alternative to time-dependent density functional theory (TD-DFT) with the same computing time scaling with system size. In particular, problematic transitions for TD-DFT such as charge-transfer, Rydberg and cyanine-like excitations were shown to be accurately described with BSE. We demonstrate here that combining the BSE formalism with the polarisable continuum model (PCM) allows us to include simultaneously linear-response and state-specific contributions to solvatochromism. This is confirmed by exploring transitions of various natures (local, charge-transfer, *etc.*) in a series of solvated molecules (acrolein, indigo, *p*-nitro-aniline, donor–acceptor complexes, *etc.*) for which we compare BSE solvatochromic shifts to those obtained by linear-response and state-specific TD-DFT implementations. Such a remarkable and unique feature is particularly valuable for the study of solvent effects on excitations presenting a hybrid localised/charge-transfer character.

## Abstract 2

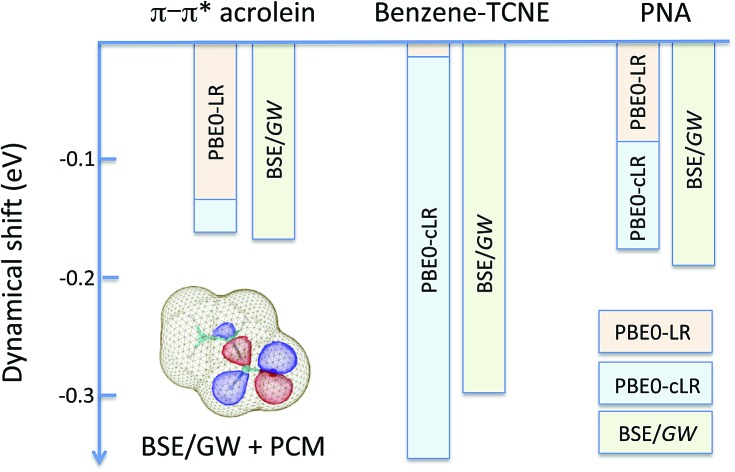
The Bethe–Salpeter formalism combined with polarizable models of the environment reconciles linear-response and state-specific contributions to solvatochromic shifts.

## 1 Introduction

The exploration of the excited-state (ES) properties of chemical systems certainly stands as a central question in theoretical chemistry. Indeed, ES phenomena govern many applications such as solar energy conversion, photocatalysis, light-emission or optical information storage. Further, while experimental characterisations can provide reference absorption and/or emission spectra, they are less suited to obtain some key information, *e.g.*, ES geometries, nature of the excitation (localised, charge-transfer, *etc.*) or time evolution of hot electrons. Such a need for quantum mechanical formalisms allowing us to study realistic systems certainly explains the formidable popularity of time-dependent density functional theory (TD-DFT)^1^,^2^ that can be used to study the optical properties of systems comprising up to a few hundred atoms, thanks to a (formal) 
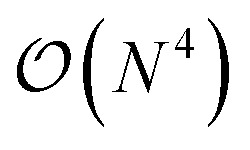
 scaling with system size. Further, the availability of analytical TD-DFT derivatives^3^–^7^ together with the extension of efficient continuum models, such as the polarisable continuum model (PCM),^8^,^9^ to TD-DFT^10^–^15^ has dramatically helped in bridging the gap between quantum simulations and realistic systems, by respectively allowing us to explore ES potential energy surfaces and to take into account the impact of the surroundings. In TD-DFT, the coupling with the PCM was initially performed within a linear-response (LR) formalism,^10^,^11^ that is, using the electronic transition density for including solvent effects. While such a LR model is generally accurate for describing the local ES, it is less suited for the charge-transfer (CT) ES, in which a large reorganisation of the electron density occurs. To tackle such an ES, state-specific (SS) PCM-TD-DFT models, in which the solvatochromic effects depend on the total electronic density of the ES, have been developed.^7^,^13^–^15^ At this stage, let us point out that, in using these SS models, one can encounter some cases for which the exact details of the chosen SS approach as well as the selected exchange-correlation functional have a very large impact on the results, especially when self-consistent iterative methods are selected.^7^,^16^–^18^ In addition, as first pointed out by Corni *et al.*,^19^ who used a simple formal model explicitly including two states for the solute and two solvent *macrostates*, there is a need to simultaneously account for both LR and SS effects. However, to date, only the *ad hoc* sum of both LR and SS terms, determined in the context of a corrected linear response (cLR) approach,^13^ was proposed in a TD-DFT context.^20^ Alternatively one can turn towards single-reference electron-correlated wavefunction approaches, such as ADC(2), CC2, CCSD or SAC-CI, that have all been coupled to continuum models,^21^–^30^ but these models imply a significantly increased computational effort compared to TD-DFT. In this framework, we underline that the importance of the inclusion of both LR and SS effects was also clearly underlined by Lunkenheimer and Köhn in their work describing the coupling of the ADC(2) theory to a continuum approach of solvation effects.^26^

As another alternative to TD-DFT, the Bethe–Salpeter equation (BSE) formalism^31^–^35^ has been recently experiencing a growing interest in the study of molecular systems due to its ability to overcome some of the problems that TD-DFT is facing, including charge-transfer^36^–^44^ and cyanine-like^45^,^46^ excitations, while preserving the same 
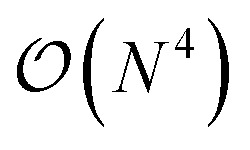
 scaling in its standard implementations. Extensive benchmark studies on diverse molecular families have been performed,^47^–^53^ demonstrating that excellent agreement with higher-level many-body wavefunction techniques, such as coupled-cluster (CC3) or CASPT2, could be obtained for all types of transitions, provided that they do not present a strong multiple-excitation character. Singlet–triplet transitions constitute the only notable exception as they may present the same instability problems with BSE and TD-DFT.^51^,^52^ We have recently reviewed the differences between BSE and TD-DFT formalisms in a chemical context, and we refer the interested reader to that original work for more details.^35^

As compared to TD-DFT, the BSE formalism relies on transition matrix elements between occupied and virtual energy levels calculated at the *GW* level, where *G* and *W* stand for the one-body Green's function and the screened-Coulomb potential. These *GW* energy levels, including HOMO and LUMO frontier orbital energies, were shown to be in much better agreement with reference wavefunction calculations as compared to standard DFT Kohn–Sham (KS) eigenvalues. Namely, *GW* HOMO and LUMO energies can be directly associated with the ionisation potential (IP) and electronic affinity (AE). Further, the TD-DFT exchange-correlation kernel matrix elements in the occupied-to-virtual transition space are replaced within the BSE formalism by matrix elements involving the screened Coulomb potential interaction between the hole and the electron.

**Fig. 1:**
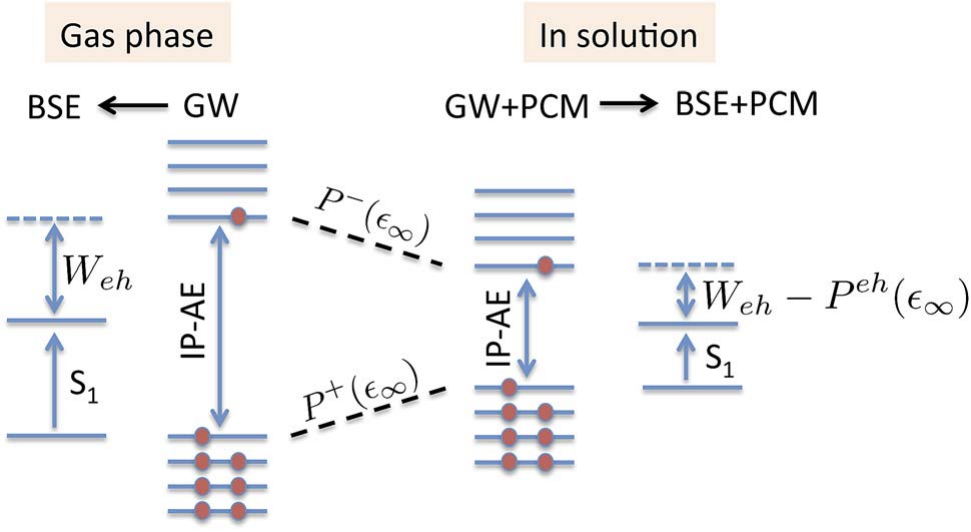
Schematic representation of the state-specific contribution to solvatochromic shifts within the (BSE/*GW* + PCM) formalism. The *GW* energy levels are first renormalised by the SS solvent-induced polarisation energies *P*_*a*_^–^(*ε*_∞_) and *P*_*i*_^+^(*ε*_∞_) for electrons (occupied) and holes (virtual), respectively. Further, the strength of the screened electron–hole interaction *W*_eh_ is also renormalised, namely reduced by a quantity that we label here *P*^eh^(*ε*_∞_), thanks to the additional screening provided by the PCM. Ground-state polarisation effects associated with the slow (*ε*_0_) degrees of freedom are incorporated at the initial (DFT + PCM) level (not represented here).

The coupling of the *GW* and PCM formalisms was recently achieved,^54^ within an integral equation formalism^9^ (IEF-PCM) implementation including the so-called non-equilibrium (neq) effects related to the separation of the solvent response into its “fast” electronic and “slow” nuclear contributions. The electron and hole polarisation energies, namely the shifts of the ionisation potential and electronic affinity from the gas phase to solution, were very well reproduced with *GW* taking as a reference standard ΔSCF + PCM calculations where total energy calculations of the solvated ions and neutral species were performed at the DFT or CCSD levels. Similar studies were also performed adopting discrete polarisable models to study organic semiconductors and complexes of interest for optoelectronic applications.^55^–^58^ As central (*GW* + PCM) formalism features, we underline that the polarisation energies for all occupied (*P*_*i*_^+^) and virtual (*P*_*a*_^–^) energy levels can be calculated, not only those associated with frontier orbitals, and that these polarisation energies are SS. Such polarisation energies, of the order of an eV in the case of water solvated nucleobases,^54^ dramatically reduce the HOMO–LUMO gap as compared to its gas phase value (see Fig. 1).

**Fig. 2:**
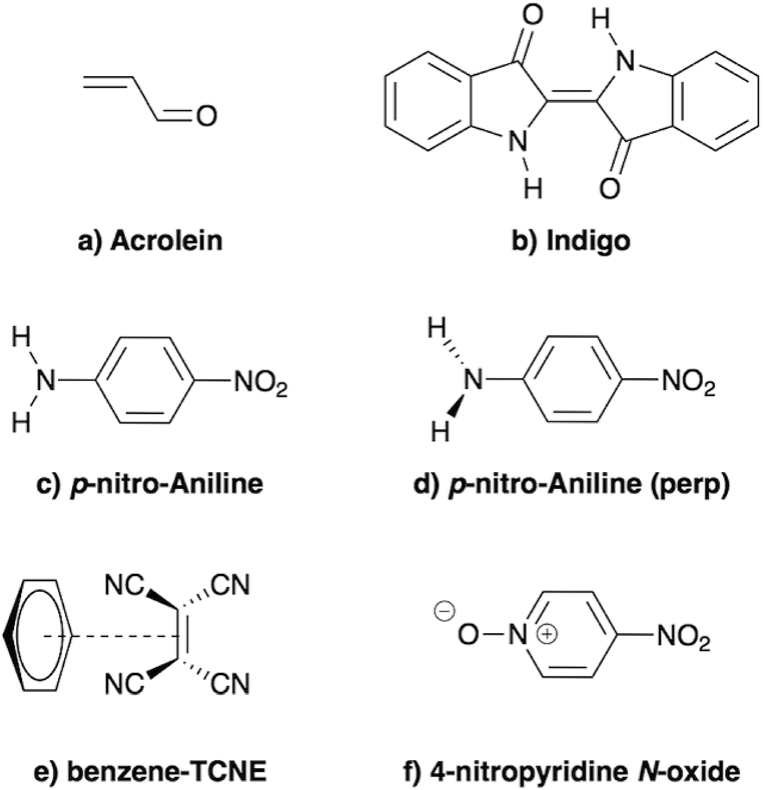
Schematic representation of the molecules and complexes studied: (a) acrolein (A), (b) indigo (I), (c) *para*-nitroaniline (PNA), (d) “twisted” *para*-nitroaniline (PNA_perp_), (e) the donor–acceptor benzene–tetracyanoethylene (B-TCNE) complex and (f) the 4-nitropyridine *N*-oxide solvatochromic probe.

In the present study, we demonstrate that the BSE formalism combined with the PCM in a non-equilibrium formulation intrinsically combines the LR and SS solvatochromic contributions associated with the effect of the polarisable environment. Such a remarkable feature hinges in particular on the proper inclusion of dynamic polarisation energies not only for the occupied and virtual energy levels, calculated within the *GW* formalism, but also for the screened Coulomb electron–hole interaction. This renormalisation by polarisation of energy levels and electron–hole interactions leads to the SS contribution to solvatochromic shifts, in addition to the familiar transition matrix elements stemming from the LR contributions. This simultaneous account of both LR and SS effects was, to the best of our knowledge, only achieved using an *a posteriori* sum of the two terms in the context of the computationally more expensive ADC(2)/COSMO approach,^26^ and more recently with a TD-DFT/discrete polarizable scheme.^20^ Here, the obtained (BSE + PCM) solvatochromic shifts are computed for paradigmatic transitions in acrolein, indigo, *p*-nitro-aniline (PNA), a small donor–acceptor complex and a solvatochromic probe (see Fig. 2), and are compared to the sum of the shifts obtained at the TD-DFT level within the standard LR and SS implementations of the PCM, respectively.

## 2 Formalism

We will not detail here the BSE formalism^31^–^34^,^59^–^61^ for which several reviews are available.^35^,^62^,^63^ We restrict ourselves to highlight the important points that allow understanding the merging with the PCM formalism. Since the BSE approach requires as an input the screened Coulomb potential *W* and accurate occupied/unoccupied {*εGWi*/*a*} energy levels calculated at the *GW* level, we start by a short introduction to the Green's function *GW* formalism^62^,^64^–^67^ from which the BSE approach derives.

### 2.1 *GW* formalism

The *GW* approach belongs to the family of many-body perturbation theories (MBPT) that takes as central quantity the Green's function (*G*) instead of, *e.g.*, the charge density within DFT. In its time-ordered formulation, the Green's function *G* reads:1
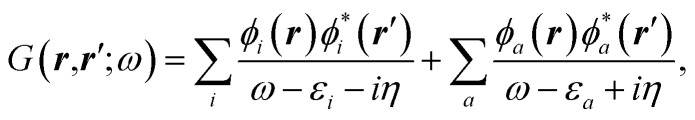
where {*ε*_*i*_,*ε*_*a*_} are the “true” electronic energy levels as defined experimentally in a direct/inverse photoemission experiment. The small positive infinitesimal *η* controls the proper analytic properties of *G* in the complex energy plane.^67^ More precisely, we define: *ε*_*a*_ = *E*(*N* + 1, *a*) – *E*(*N*, 0) for “virtual” energy levels and *ε*_*i*_ = *E*(*N*, 0) – *E*(*N* – 1, 0) for “occupied” energy levels, where *E*(*N* + 1, *a*) is the total energy of the (*N* + 1)-electron system in its *a*^th^ quantum state, *E*(*N* – 1, *i*) the total energy of the (*N* – 1)-electron system in its *i*^th^ quantum state, and *E*(*N*, 0) the *N*-electron system ground-state energy. {*φ*_*i*_, *φ*_*a*_} are called “Lehman amplitudes”. It can be formally shown that *G* verifies a simple Dyson equation:2
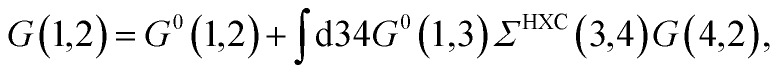
with, *e.g.*, 1 = (***r***_1_,*t*_1_) a space-time coordinate. *G*^0^ is the independent-electron Green's function and the “self-energy” operator *Σ*^HXC^ contains all electron–electron interactions (Hartree plus exchange and correlation). Plugging the expression for *G* into the Dyson equation leads to a familiar eigenvalue equation for the (photoemission) excitation energies defined here above, namely:3
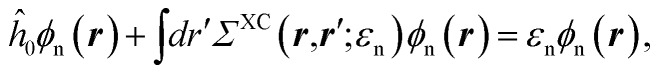
where the *h*^^^_0_ Hamiltonian contains the kinetic, ionic and classical Hartree operators. Such an equation is formally equivalent to the DFT KS equation, but with an exchange-correlation “potential” that is both non-local and energy-dependent.

While eqn (3) is exact, an expression for *Σ*^XC^ should be defined. The *GW* formalism provides an approximation for *Σ*^XC^ to first order in the screened Coulomb potential *W*, with:4
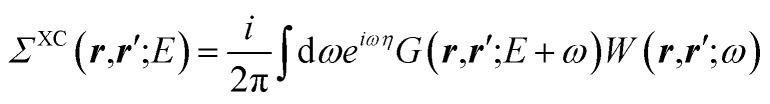

5
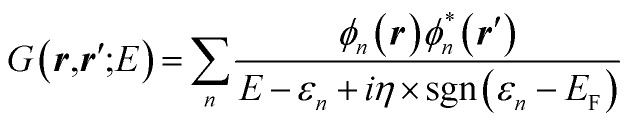

6
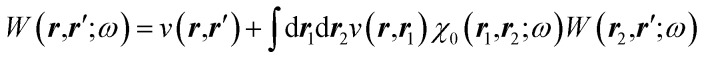

7
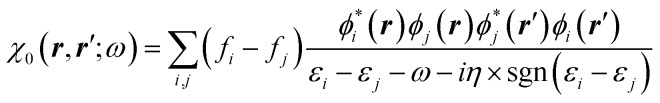
where *v*(***r***,***r***′) is the bare Coulomb potential, *χ*_0_ the independent-electron susceptibility and *W* the screened Coulomb potential. *E*_F_ is the energy of the Fermi level. {*f*_*i*/*j*_} are occupation numbers and the input {*φ*_*n*_, *ε*_*n*_} eigenstates are typically KS eigenstates that will be corrected within the *GW* formalism.

The *GW* eigenvalues have been shown in many benchmark studies on gas phase molecular systems to be significantly more accurate than KS or Hartree–Fock eigenstates, providing, *e.g.*, frontier orbital energies within a few tenths of an eV with respect to reference CCSD(T) calculations.^68^–^72^ For the sake of illustration, our gas phase ionization potential (IP) and electronic affinity (EA) are 10.35 eV (–*εGW*HOMO) and 0.68 eV (+*εGW*LUMO) respectively for acrolein within our *GW* approach, comparable to 10.01 eV and 0.70 eV within CCSD(T) for the same atomic basis (cc-pVTZ) and the same geometry (see the ESI†). In practice, *GW* calculations proceed traditionally by correcting input KS eigenstates, substituting the *GW* self-energy contribution to the DFT exchange-correlation potential:8
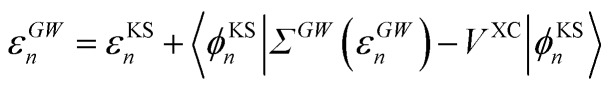



The obtained *εGWa*/*i* eigenvalues and the screened Coulomb potential *W* serve as input quantities for the BSE excitation energy calculation.

### 2.2 Bethe–Salpeter equation

While TD-DFT starts from the evaluation of the density–density susceptibility *χ*(12) = ∂*n*(1)/∂*U*^ext^(2) that measures the variation of the charge-density with respect to an external local perturbation, the excitation energies within BSE are obtained through the poles of a generalized susceptibility *L*(1234) = ∂*G*(12)/∂*U*^ext^(34), where *U*^ext^(34) is a non-local external perturbation. Deriving the Dyson equation for *G* (eqn (2)) leads in particular to the introduction of the (∂*Σ*^XC^/∂*G*) derivative, the analog to the exchange-correlation kernel within DFT. In the standard BSE/*GW* formalism, we consistently assume the *GW* approximation for *Σ*^XC^. Expressing then the 4-point susceptibility *L* in the transition space between occupied and unoccupied one-body eigenstates yields a standard linear algebra eigenvalue representation:9
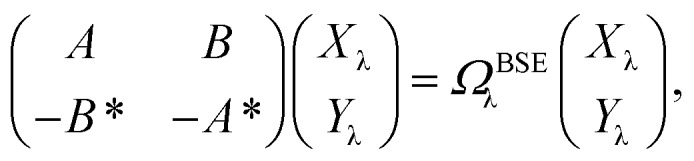
where *Ω*BSEλ represents the BSE excitation energies. *X*_λ_ represents the components of the two-body electron–hole *ψ*_λ_(***r***_e_,***r***_h_) eigenstates over the *φ*_*i*_(***r***_h_)*φ*_*a*_(***r***_e_) transition basis, where (*i*,*a*) index (occupied, virtual) eigenstates, while *Y*_λ_ gathers the *φ*_*i*_(***r***_e_) *φ*_*a*_(***r***_h_) de-excitation components. Such a linear algebra representation in the transition space resembles the standard TD-DFT formalism within the so-called Casida's formulation^1^ but with modified matrix elements. For the resonant block, one obtains:

10 *A*BSE*ai*,*bj* = *δ*_*ab*_*δ*_*ij*_(*εGWa* – *εGWi*) – *ab*|*W*|*ij* + *ai*|*bj*

with

11 *ab*|*W*|*ij* = *φ*_*a*_(***r***)*φ*_*b*_(***r***)*W*(***r***,***r***′)*φ*_*i*_*(****r***′)*φ*_*j*_(***r***′)

12 *ai*|*bj* = *φ*_*a*_(***r***)*φ*_*i*_(***r***)*v*(***r***,***r***′)*φ*_*b*_(***r***′)*φ*_*j*_(***r***′)

where *εGWa*/*i* are the *GW* unoccupied/occupied energy levels and *W* the screened Coulomb potential. The (*εGWa* – *εGWi*) energy differences replace the TD-DFT KS (*ε*KS*a* – *ε*KS*i*) energy differences between virtual and occupied states, while the *W* matrix elements can be interpreted as electron–hole interaction terms through the screened Coulomb potential. {*φ*_*i*/*a*_} are the starting KS one-body eigenstates that are not corrected in the *GW* implementation selected here (see below).

### 2.3 Merging with the PCM formalism

The merging of the *GW* formalism with the PCM is described in detail in ref. 54. The seminal point is that the solvent reaction field can be straightforwardly incorporated into the screened Coulomb potential *W* that accounts for both the polarisability of the solute and that of the solvent when combined with the PCM. Under the assumption that the solute and solvent eigenstates do not spatially overlap, it can be shown that the screened Coulomb potential *W* is changed from its gas phase value:13
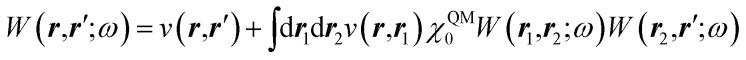
to its condensed phase expression:

14 *W* = ** + *χ*QM0*W*

15 ** = *v* + *vχ*PCM0**

where we have dropped the frequency and space variables in the second equation for conciseness. *χ*QM0 is the gas phase independent-electron susceptibility of the quantum subsystem to be solvated, while *χ*PCM0 is that of the PCM solvent. The modified ** potential is thus the bare Coulomb potential renormalized by the solvent reaction field, equivalent to:

16 ** = *v* + *vχ*^PCM^*v*

where *χ*^PCM^ is the full (interacting) susceptibility of the solvent. The quantity [*vχ*^PCM^*v*] (***r***_1_,***r***_2_) represents the reaction field (*v*^reac^) generated in ***r***_2_ by the PCM surface charges developed in response to a unity point-charge added in ***r***_1_, with (***r***_1_,***r***_2_) located in the cavity carved in the solvent to accommodate the solute. Details of our implementation using localised Gaussian bases and the Coulomb-fitting resolution-of-identity (RI-V) approach can be found in ref. 54 for the merging of *GW* with the PCM and ref. 56 and 58 for the merging with a discrete polarisable model.

Importantly, in the present non-equilibrium formulation of the (BSE/*GW* + PCM) implementation, the reaction field that modifies *W* is associated with the “fast” electronic excitations, namely with the *ε*_∞_ dielectric constant (*e.g.*, *ε*_∞_ = 1.78 in water) that is the low-frequency optical dielectric response. The inclusion of the “slow” response of the solute (given by, *e.g.*, *ε*_0_ = 78.35 for water) is accounted for in the preliminary ground-state DFT + PCM(*ε*_0_) run that serves as a starting point for *GW* and BSE calculations, namely in the construction of *χ*_0_ and *G* in eqn (5) and (7). The flow of calculations can then be summarised as follows:

(1) Calculation of input KS {*ε*_*n*_, *φ*_*n*_} eigenstates within a DFT + PCM(*ε*_0_) scheme. These eigenstates contain ground-state solvation effects,

(2) Calculation of the “fast” reaction field *v*^reac^(*ε*_∞_) in an auxiliary basis representation (see ref. 54, 56 and 58) that is incorporated inside the screened Coulomb potential *W* following eqn (5) and (7),

(3) Correction of the {*ε*_*n*_} KS eigenstates, that include “slow” polarisation effects, with the *GW* self-energy operator that contains the “fast” *v*^reac^(*ε*_∞_) reaction field in order to yield the *GW* + PCM neq {*ε*_*n*_^*GW*+PCM^} energy levels,

(4) Resolution of the BSE excitation energy eigenvalue problem with the {*ε*_*n*_^*GW*+PCM^} eigenstates and the *W* and ** potentials that include *v*^reac^(*ε*_∞_) = *vχ*^PCM^(*ε*_∞_)*v*.

### 2.4 LR and SS solvatochromic contributions

The renormalisation of *v* into *v* + *v*^reac^(*ε*_∞_) and the resulting change Δ*W*, accounting for switching on the fast *ε*_∞_ solvent response, leads to modifying eqn (10) as follows:17a
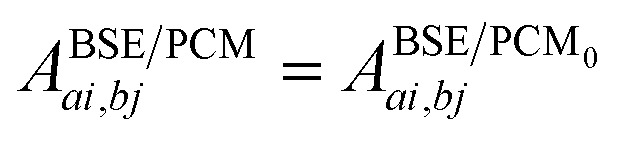

17b +*δ*_*ab*_*δ*_*ij*_Δ(*εGW*/PCM*a* – *εGW*/PCM*i*) – *ab*|Δ*W*|*ij*

17c +*ai*|*vχ*^PCM^(*ε*_∞_)*v*|*bj*,

where *A*^BSE/PCM_0_^ is the BSE Hamiltonian that includes the ground-state charges only, namely built with *ε*_∞_ = 1. Correspondingly, Δ*εGW*/PCM*a*/*i* corresponds to the shift of the *GW* energy levels when switching on the fast solvent response, that is, 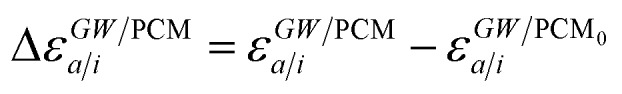. Such a decomposition allows a direct correspondence with the solvatochromic shifts calculated within TD-DFT in both the LR and SS formulations.

As the easiest identification, the 3^rd^ line contribution (eqn (17c)) straightforwardly corresponds to LR reaction field matrix elements, *i.e.*, to transition density polarisation effects. More explicitly, *ai*|*vχ*^PCM^*v*|*bj* describes the action on *φ*_*b*_(***r***′)*φ*_*j*_(**r**′) of the reaction field 
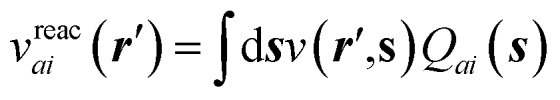
, where 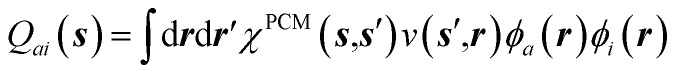 is the surface charge generated by the PCM susceptibility *χ*^PCM^(*ε*_∞_) in response to the field generated by the transition density *φ*_*a*_(***r***)*φ*_*i*_(***r***). In the original notations of ref. 10, such matrix elements are strictly equivalent to the *B*f*ai*,*bj* = *ai*|*K*^f^|*bj* linear response terms (eqn (31) in ref. 10) where *K*^f^ is the “fast” reaction potential integral operator. As such, the BSE + PCM formalism includes the LR solvent contributions.

Let us now demonstrate that the second line (eqn (17b)) recovers the SS contribution. Part of the demonstration relies on the specificity of the *GW* + PCM formalism that captures accurately SS dynamic polarisation energies, namely:18a
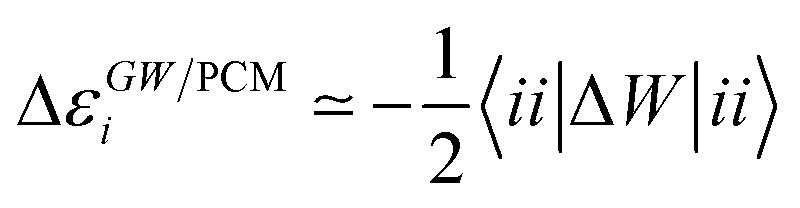

18b
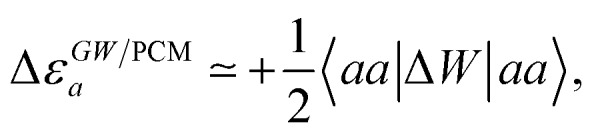
with positive (negative) shifts for occupied (virtual) levels since Δ*W* corresponds to a reduced interaction. This was demonstrated numerically in ref. 54 and justified in Appendix A. As such, the term of eqn (17b), that we label Δ*A*SS*ai*,*bj*, reads:19
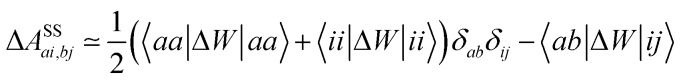



In the case of a “pure” transition between levels (*i*) and (*a*), one straightforwardly obtains:20
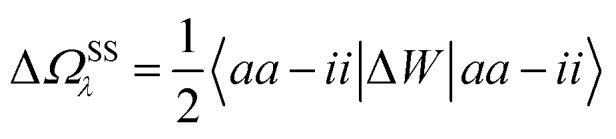
where (*aa* – *ii*) represents the unrelaxed variation Δ*ρ*_*i*→*a*_ of the charge density between the excited and the ground states. Identifying Δ*W* to the reaction field *vχ*^PCM^(*ε*_∞_)*v* as justified in Appendix B, we recognise the action on Δ*ρ*_*i*→*a*_ of the reaction field 
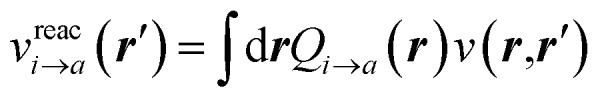
, where *Q*_*i*→*a*_ (***r***) is the surface charge generated by the PCM susceptibility *χ*^PCM^(*ε*_∞_) in response to the field generated by Δ*ρ*_*i*→*a*_ itself, with the (1/2) factor indicating that it is a self-interaction term. This corresponds to the SS expression within TD-DFT for the solvatochromic shift, namely with the notations of ref. 12:21
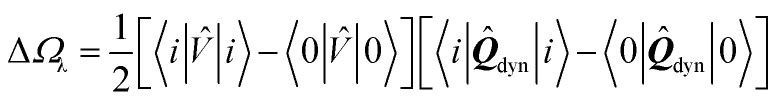
where here *i* represents the *i*^th^ ES. Physically, the terms in eqn (17b) represent the quantum mechanical version of the classical solvation energy associated with the interaction of a charge redistribution (dipolar, quadrupolar, *etc.*) with the polarisable medium, where this charge redistribution corresponds to the charge variation between the ground and the excited states.

We now turn to the case of general BSE electron–hole eigenstates 
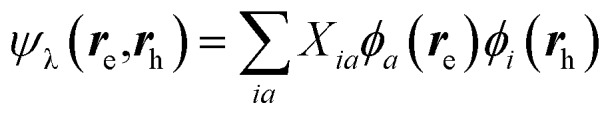
 assuming for simplicity the TDA approximation. Hole and electron densities are now described by a correlated codensity *ρ*_λ_(***r***_e_,***r***_h_) = |*ψ*_λ_(***r***_e_,***r***_h_)|^2^ that cannot be expressed in terms of individual eigenstate densities as in the simple two-level model. This however does not affect the central result that the screened Coulomb potential *W*, and thus the electron–hole interaction:22
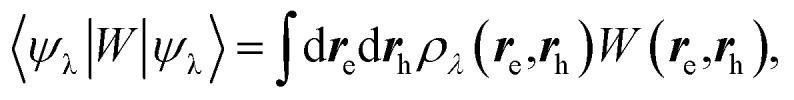
are properly renormalized by the reaction field. Further, in the presence of the PCM, the BSE electron–hole Hamiltonian of eqn (10) is first dressed with the reaction field operator *v*^reac^ and then fully rediagonalised. As such, beyond first-order perturbation theory, the (BSE + PCM) eigenstates and codensities are relaxed with respect to the presence of the polarisable medium and the variation from gas to solvent of the electron–hole interaction results from the variation of both *ρ*_λ_ and *W*. It should be emphasized however that in the present implementation, the molecular orbitals and corresponding codensities are not relaxed with respect to the change in QM charge density upon excitation. Namely, the so-called *Z*-matrix relaxation effects are here neglected. From this point of view, the present BSE/PCM methodology is at that stage in analogy with the unrelaxed density (UD) approximation used in the Vertical Excitation Method (VEM) approach (VEM-UD).^7^,^15^

Concerning now the contribution from the *GW* electron and hole quasiparticle energies, we obtain, considering *e.g.* the unoccupied (electron) energy levels *εGW*/PCM*a*:23
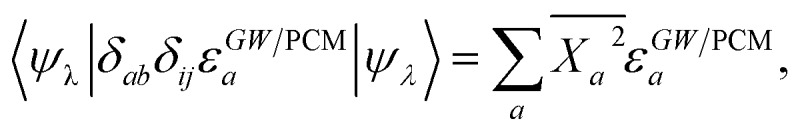
with 
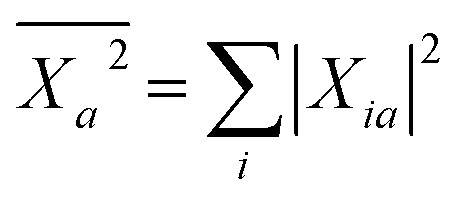
. The variation of the electron and hole energies from the gas to the solvent thus stems from the variation of the *X*_*ia*_ coefficients (relaxation effects) and from that of the individual *εGW*/PCM*i*/*a* energy levels, namely their related polarization energy Δ*εGWi*/*a*. In the correlated electron–hole BSE description, the variation of the electronic energy levels contributes to the excitation energy solvatochromic shift through a weighted average of the individual level polarization energy.

## 3 Computational details

The geometries of all compounds have been obtained at the MP2/6-311G(2d,2p) level in the gas-phase, but for the benzene/TCNE complex the geometry was taken from ref. 73 (see the ESI†). Our *GW* and BSE calculations are performed with the Fiesta package^44^,^47^,^53^ that implements these formalisms with Gaussian basis sets and the Coulomb-fitting RI-V approach.^74^ Input KS eigenstates are generated with the NWChem package^75^ at the PBE0/cc-pVTZ level.^76^–^78^ The corresponding cc-pVTZ-RI auxiliary basis set^79^ is used in the many-body RI-V calculations. For improved accuracy and removal of most of the dependency on the selected DFT functional, our *GW* calculations are performed at the partially self-consistent ev*GW* level^68^,^70^,^72^,^80^,^81^ with *GW*-updated eigenvalues and frozen eigenvectors.

Within the present neq approach, the starting DFT calculations are performed in combination with the COSMO formalism^82^ as implemented in NWChem, using the (*ε*_0_) dielectric constant associated with the (slow) nuclear and electronic degrees of freedom (*e.g.*, *ε*_0_ = 78.39 for water). As such, the KS eigenstates used to build our electronic excitations carry information regarding the ground-state polarisation effects. In a second-step, the screened Coulomb potential used in the *GW* and BSE calculations, that describes fast electronic excitations out of the ground-state, is “dressed” with the reaction field generated with the (fast) electronic dielectric response in the low-frequency limit (*e.g.*, *ε*_∞_ = 1.78 for water). In *GW* and BSE, the reaction field is described at the full IEF-PCM level, following the implementation detailed in ref. 54.

Our BSE calculations are compared to TD-DFT calculations performed with the Gaussian16 package^83^ using the IEF-PCM non-equilibrium implementation and the same cc-pVTZ atomic basis set. The LR shifts have been obtained with the default Gaussian16 implementation,^10^,^11^ whereas the SS shifts have been determined with the so-called corrected LR (cLR) formalism,^13^ that is a perturbative approach. For the TD-DFT calculations we selected the same PBE0 functional, complemented in the case of CT transitions with calculations performed with the range-separated hybrid CAM-B3LYP functional,^84^ known to more accurately describe CT transitions in the TD-DFT context. The character of the electronic transitions in TD-DFT has been determined by estimating the effective electronic displacement induced by the excitation using the *Γ* index.^85^,^86^

In order to differentiate static and dynamic contributions within our approach, we make use of a ground state frozen polarization excitation energy (*Ω*_0_),^15^,^19^ obtained by considering that the polarisable medium does not respond to the fast electronic excitation, namely by setting *ε*_∞_ = 1 while keeping the correct static dielectric constant of the solvent *ε*_0_. As such, labelling *Ω* the final BSE excitation energies, accounting for both static and dynamic PCM responses, the quantity (*Ω* – *Ω*_0_) quantifies the impact of *switching on* the fast PCM(*ε*_∞_) response. We underline that our BSE calculations are performed beyond the Tamm–Dancoff approximation (TDA), that is, include the full BSE matrix. However, the inclusion of contributions from de-excitation processes complicates the simple analysis provided above concerning the LR and SS contributions with a clear distinction between *ab*|*W*|*ij* terms, namely the coupling between density terms, and *ai*|*v*^reac^|*bj* contributions, that is the coupling between transition dipoles. Beyond TDA, we can decompose the expectation value *Ψ*_BSE_|*H*_BSE_|*Ψ*_BSE_ into24a
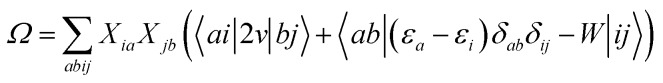

24b
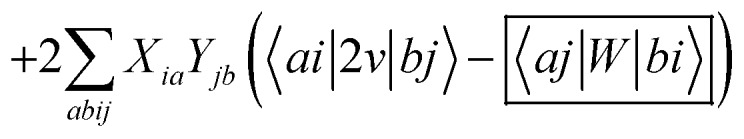

24c
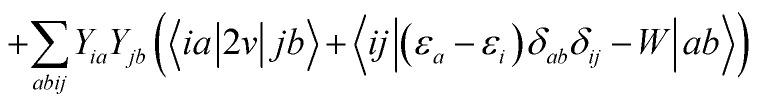
where we have dropped the *λ* excitation index for simplicity and where the factor 2 in front of the bare Coulomb potential indicates singlet transition energies. In such a decomposition, all terms involving occupied-virtual orbital products, including the (boxed) *aj*|*W*|*bi* term, contribute to the LR response, whereas the remaining matrix elements contribute to the SS response.

## 4 Results

To illustrate the methodology and to confirm that the PCM-BSE formalism provides both LR and SS shifts, we perform calculations on a series of standard test molecules (Fig. 2), providing comparison with TD-DFT/PCM calculations conducted with the standard LR and SS (cLR) response formalisms. We start with transitions that have a rather local character and hence are expected to be better described within TD-DFT by the LR formalism (Subsection 4.1), before focusing in a second Subsection 4.2 on transitions having a strong CT character that requires the SS formalism. Eventually, in Subsection 4.3, we evidence that some systems may require both LR and SS contributions, highlighting the merits of the (BSE + PCM) formalism that can treat all systems on an equal footing.

### 4.1 Local ES: dominating LR contributions

We start our analysis with the well-known cases of acrolein and indigo (Fig. 2a and b). Acrolein is characterised by a negative solvatochromism for the lowest n–π* (*A*′′) transition but a positive solvatochromism of the lowest π–π* (*A*′) excitation. Experimental data are available in both the gas phase and water,^87^,^88^ and acrolein has been used as a benchmark system for studying continuum solvation models in conjunction with several levels of theory, *e.g.*, TD-DFT,^12^,^13^,^15^ ADC(2),^26^ SAC-CI,^21^,^23^ and CCSD.^22^,^25^,^27^ Hybrid two-layer QM/MM^88^,^89^ or three-layer QM/MM/PCM^90^ calculations, combining TD-DFT with an atomistic force field description of the explicit solvent, have also been used to understand the *pros* and *cons* of the PCM model for water, a protic medium well-known to be challenging for continuum approaches.

Our data are compiled in Table 1. The gas phase (*Ω*_g_) and solvated (*Ω*) theoretical and experimental excitation energies are provided, together with the overall shift (*Ω* – *Ω*_g_). In our non-equilibrium formalism, the shift is decomposed into a ground-state static contribution (*Ω*_0_ – *Ω*_g_) and a dynamic one (*Ω* – *Ω*_0_) that is itself partitioned into LR and SS contributions. Within TD-DFT, the LR and SS (cLR) shifts are obtained as two separate calculations, whereas within BSE, this decomposition is obtained by partitioning (see above) the non-equilibrium BSE/*GW* + PCM shift. Wavefunction approaches, such as ADC(2) or CCSD, can also be used to provide both contributions simultaneously^26^ or separately^25^ and we give some literature examples in Table 1.

**Table 1:** Lowest singlet excitation energies for acrolein and indigo in the gas phase (*Ω*_g_) and in water (*Ω*), combining TD-DFT or BSE with neq PCM (*ε*_0_ = 78.39, *ε*_∞_ = 1.78 for water). The *Ω*_0_ energies are obtained by setting *ε*_∞_ = 1, namely accounting only for ground-state polarisation effects with no additional surface charges induced by the excitation. The TD-PBE0 (*Ω* – *Ω*_0_) shifts are provided using either the LR or SS (cLR) formalisms. The (*Ω* – *Ω*_0_) BSE shifts are also partitioned into LR and SS contributions following the analysis in Section 3. All values are in eV

	*Ω*_g_	*Ω*(*Ω* – *Ω*_g_)	*Ω*_0_ – *Ω*_g_	*Ω* – *Ω*_0_		Ref.
				LR	SS	
Acrolein n–π* in water						
TD-PBE0 (LR)	3.599	3.785(+0.186)	+0.189	–0.003		This work
TD-PBE0 (cLR)	3.599	3.736(+0.137)	+0.189		–0.052	This work
BSE	3.736	3.988(+0.252)	+0.232	–0.011	+0.031	This work
CC3	3.74					This work
CCSDR(3)/MM	3.81	4.08(+0.27)				91
SAC-CI	3.85	3.95(+0.10)				21
CCSD	3.94	4.14(+0.20)				22
ADC(2)	3.69	3.86(+0.17)^*a*^				26
CCSD (LR)	3.88	4.10(+0.22)				25
CCSD (SS)	3.88	4.05(+0.17)				25
Exp.	3.69	3.94(+0.25)				87 and 88

Acrolein π–π* in water						
TD-PBE0 (LR)	6.383	6.174(–0.209)	–0.073	–0.136		This work
TD-PBE0 (cLR)	6.383	6.281(–0.102)	–0.073		–0.029	This work
BSE	6.498	6.214(–0.284)	–0.112	–0.163	–0.004	This work
CC3	6.82					This work
CCSDR(3)/MM	6.73	6.22(–0.51)				91
SAC-CI	6.97	6.75(–0.22)				21
CCSD	6.89	6.54(–0.35)				22
ADC(2)	6.79	6.40(–0.39)^*b*^				26
CCSD (LR)	6.80	6.39(–0.41)				25
CCSD (SS)	6.80	6.54(–0.26)				25
Exp.	6.42	5.89(–0.53)^*a*^				87 and 88

**Indigo in water**						
TD-PBE0 (LR)	2.304	2.160(–0.144)	–0.068	–0.076		This work
TD-PBE0 (cLR)	2.304	2.229(–0.075)	–0.068		–0.007	This work
BSE	2.259	2.047(–0.212)	–0.122	–0.082	–0.008	This work
Exp.	2.32	2.04(–0.19)^*c*^				92, 93, 94, 95, 96, 97 and 98

^*a*^In the breakdown approach used in that work, the SS contribution is +0.17 eV and the LR contribution is negligible.

^*b*^In the breakdown approach used in that work, the SS contribution is –0.21 eV and the LR contribution is –0.18 eV.

^*c*^In ethanol, the most polar protic solvent in which indigo is soluble experimentally.

The acrolein n–π* transition in the gas phase (*Ω*_g_) is found to be located at 3.60 eV and 3.74 eV within TD-PBE0 and BSE respectively, in good agreement with the CC3 value of 3.74 eV, as well as with previous wavefunction estimates and experiment. The analysis of the intermediate (*Ω*_0_ – *Ω*_g_) and total (*Ω* – *Ω*_g_) solvatochromic shift indicates that the positive solvatochromism for this transition is entirely dominated by ground-state effects and that the additional shift associated with the fast optical excitation is negligible. TD-PBE0 and BSE calculations performed on top of the DFT + PCM(*ε*_0_) ground-state yield similar *Ω*_0_ – *Ω*_g_ shifts, namely +0.19 eV and +0.23 eV, respectively. Concerning the effect of switching *ε*_∞_ (1.78 in water), both TD-PBE0+PCM, within LR or cLR, and BSE + PCM yield very small additional shifts, ranging from –0.05 eV (cLR) to +0.02 eV (BSE). The BSE computed shift of +0.25 eV turns out to be in close agreement with the experimental values (+0.25 eV), as well as with previous wavefunction approaches (see Table 1).

While the n–π* transition does not allow us to clearly discriminate between LR and SS responses, a more interesting test of the effect of the fast response (*ε*_∞_) polarisable environment comes with the higher-lying π–π* transition. For this transition, the reaction field associated with the optical excitation, namely *v*^reac^(*ε*_∞_), leads to a shift that is larger than the one associated with ground-state solvation charges. In the gas phase, the BSE transition energy (6.50 eV) is reasonably close to the CC3 (6.82 eV) and experimental (6.42 eV) values. The most salient feature is that within TD-DFT, only the LR scheme can significantly contribute to the redshift, while the cLR approach fails to deliver any sizeable solvation effect, with (*Ω* – *Ω*_0_) being equal to –0.14 eV for the former model, and –0.03 eV for the latter. This effect was expected for a local π–π* transition not involving a strong density reorganisation between the two states: the LR-PCM-TD-DFT is more suited as it captures the dominating contributions originating from the transition densities, that can be viewed as “dispersion-like” terms.^19^ At the CCSD level, the results of Caricato^25^ also demonstrated that the LR contribution is dominant; in that work the decomposition of the total response into various contributions was not performed, but rather two different models have been applied as in TD-DFT. We also underline that in their ADC(2) study, Lunkenheimer and Köhn also found that the LR term, negligible for the n–π* case,^26^ becomes large for the π–π* transition, though the approach used to compute the relative contributions is not straightforwardly comparable to ours. In any case, the BSE + PCM formalism, with a –0.17 eV (*Ω* – *Ω*_0_) shift, captures the correct bathochromic effect. Further, consistent with the TD-DFT calculations, we observe that the BSE shift is dominated by the LR contribution, while the SS term provides a negligible shift. This shows, consistent with our analysis of eqn (17c), that the BSE formalism correctly captures the LR response. When compared to experiment, the BSE + PCM shift remains too small, but we recall that we neglect here, as in any continuum approach, the explicit solvent–solute interactions that are known to be significant in the present case.^26^,^91^

To confirm the present observations, we consider the case of the lowest transition in indigo, a hallmark centro-symmetric dye presenting a low-lying dipole-allowed π–π* transition. This compound was studied previously at the TD-DFT level with the LR PCM model, and it was shown that this approach nicely reproduces the experimental solvatochromic shifts.^99^ With TD-DFT and the LR formalism, we found that the ground-state and dynamic polarisation effects, as measured respectively by (*Ω*_0_ – *Ω*_g_) and (*Ω* – *Ω*_0_), have the same order of magnitude, whereas the cLR correction does not lead to any significant (*Ω* – *Ω*_0_) effect, as expected for a dye in which both the ground-state and excited-state total dipoles are strictly null. As in the case of the π–π* transition in acrolein, the BSE + PCM scheme leads to a clear redshift, with in this case a good agreement with the experimental trends as well. As a matter of fact, the (*Ω* – *Ω*_0_) BSE LR (SS) shift is very close to the corresponding TD-DFT LR (SS) shift, demonstrating the relevance of the analysis and partitioning of the BSE overall (*Ω* – *Ω*_0_) difference discussed in Section 3.

### 4.2 Charge-transfer ES: dominating SS contributions

We now turn to the study of charge-transfer (CT) excitations for which the SS contribution should increase with increasing CT character. We start with the *p*-nitro-aniline (PNA) molecule (Fig. 2c), a typical push–pull system studied both theoretically^5^,^100^ and experimentally,^101^ and characterised by a low-lying CT excitation from the amine (–NH_2_) to the nitro (–NO_2_) group. Inspired by ref. 5, we first consider an artificial configuration obtained by rotating the amino group perpendicularly to the conjugated plane (PNA_perp_ in Fig. 2d) so as to produce a transition having a *pure* dark CT character. We focus on the low-lying dark CT excitation showing a dominant HOMO–LUMO character. The “standard” planar PNA molecule is studied below. Further, we consider an intermolecular CT excitation in the donor–acceptor benzene/TCNE complex (Fig. 2e),^38^,^73^ for which gas phase experimental data are available.^102^ The lowest energy excitation is a clear HOMO–LUMO transition with a strong CT character even though a small delocalization of the HOMO (LUMO) on TCNE (Benzene) leads to a non-zero oscillator strength (see *e.g.*ref. 38). Since CT transitions are poorly described by global hybrid functionals with a moderate amount of exact exchange such as PBE0, TD-DFT calculations are also performed with the CAM-B3LYP range-separated hybrid functional. Our data are compiled in Table 2.

**Table 2:** Data as in Table 1 but for the lowest CT transitions in PNA_perp_ and in the benzene–TCNE complex. The experimental value for the latter case is taken from ref. 102

	*Ω*_g_	*Ω*(*Ω* – *Ω*_g_)	*Ω*_0_ – *Ω*_g_	*Ω* – *Ω*_0_	
				LR	SS
**PNA**_**perp**_ **in water (“dark” CT excitation)**					
TD-PBE0 (LR)	3.686	3.316(–0.370)	–0.370	0.000	
TD-PBE0 (cLR)	3.686	2.861(–0.795)	–0.370		–0.425
TD-CAM-B3LYP (LR)	4.621	4.308(–0.313)	–0.312	–0.001	
TD-CAM-B3LYP (cLR)	4.621	4.038(–0.583)	–0.312		–0.271
BSE	5.112	4.399(–0.713)	–0.423	+0.015	–0.304

**Benzene–TCNE in water (“bright” CT excitation)**					
TD-PBE0 (LR)	2.157	2.081(–0.076)	–0.065	–0.011	
TD-PBE0 (cLR)	2.157	1.747(–0.410)	–0.065		–0.345
TD-CAM-B3LYP (LR)	2.944	2.876(–0.068)	–0.061	–0.007	
TD-CAM-B3LYP (cLR)	2.944	2.492(–0.452)	–0.061		–0.291
BSE	3.503	3.121(–0.382)	–0.086	–0.009	–0.287
Exp.	3.59				

While the contribution of the fast optical dielectric response (*ε*_∞_) to the solvatochromic shifts, as measured by (*Ω* – *Ω*_0_), mainly originates from the LR contribution in both acrolein and indigo, the solvent-induced dynamic shift associated with CT transitions can only be described by adopting a SS (cLR here) formalism in the TD-DFT context. This is clearly illustrated by the PNA_perp_ system where the TD-DFT LR shift is trifling, a logical consequence of the dark nature of the considered transition, whereas the SS contribution is very large, as a result of the large density reorganisation associated with excitation. Consistent with previous studies,^17^ the magnitude of the TD-DFT SS shift strongly depends on the chosen functional, and it goes from –0.42 eV to –0.27 eV upon replacing the PBE0 functional by CAM-B3LYP, which is more suited for such an excited-state. The BSE (*Ω* – *Ω*_0_) shift originates mainly from its SS component as well and lies in between the PBE0 and CAM-B3LYP values, though much closer to the latter, as expected.

The same conclusions are reached when considering the inter-molecular CT transition in the benzene–TCNE complex. First, we observe that the BSE formalism very nicely reproduces the gas phase excitation energy that is available experimentally. The TD-CAM-B3LYP calculation also provide a reasonable value, while the TD-PBE0 approach yields a much too small *Ω*_g_, a logical consequence of its lack of long-range corrections. As expected for TD-DFT, the LR approach again provides a negligible (*Ω* – *Ω*_0_) dynamic shift, while the SS formalism yields a large redshift. Again, the BSE + PCM formalism captures the correct physics, with a negligible LR contribution and a large SS contribution. Such calculation clearly demonstrates that the proposed BSE + PCM approach can also, following eqn (17b), capture SS dynamic shifts.

### 4.3 Hybrid ES

We now turn to difficult cases where neither the LR nor SS contributions can be neglected, and our results are collected in Table 3. As such, TD-DFT calculations adopting one or the other response formalisms cannot capture the entire solvatochromic shifts, though, as discussed in ref. 26 both contributions should in principle be accounted for. This is first evidenced by considering the planar (standard) PNA molecule. The much smaller disagreement between TD-PBE0 and TD-CAM-B3LYP, as compared to the PNA_perp_ case, is indeed a first indication that the CT character is here significantly reduced. As a result (see Table 3), both SS and LR contributions to the (*Ω* – *Ω*_0_) shift are large: they are of equal magnitude within TD-PBE0 while the SS contribution is larger with TD-CAM-B3LYP. Clearly, the BSE formalism simultaneously accounts for both contributions, the SS contribution being slightly larger than the LR one, consistent with the TD-DFT results. The total (*Ω* – *Ω*_0_) dynamic shift amounts to –0.18 eV and –0.27 eV when adding the LR and cLR contribution determined at TD-PBE0 and TD-CAM-B3LYP, respectively, comparable to the –0.19 eV solvatochromic shift obtained at the BSE level.

**Table 3:** Data as in Table 1 but for the transitions of mixed character

	*Ω*_g_	*Ω*(*Ω* – *Ω*_g_)	*Ω*_0_ – *Ω*_g_	*Ω* – *Ω*_0_	
				LR	SS
**PNA in water (partial CT excitation)**					
TD-PBE0 (LR)	4.202	3.802(–0.400)	–0.314	–0.086	
TD-PBE0 (cLR)	4.202	3.796(–0.406)	–0.314		–0.092
TD-CAM-B3LYP (LR)	4.513	4.105(–0.408)	–0.321	–0.087	
TD-CAM-B3LYP (cLR)	4.513	4.005(–0.508)	–0.321		–0.187
BSE	4.527	3.864(–0.663)	–0.470	–0.090	–0.102

**4-Nitropyridine *N*-oxide in benzene (mixed excitation)**					
TD-PBE0 (LR)	3.989	3.815(–0.174)	–0.048	–0.126	
TD-PBE0 (cLR)	3.989	3.797(–0.192)	–0.048		–0.144
TD-CAM-B3LYP (LR)	4.196	4.023(–0.173)	–0.033	–0.140	
TD-CAM-B3LYP (cLR)	4.196	4.109(–0.087)	–0.033		–0.054
BSE	3.966	3.658(–0.267)	–0.001	–0.193	–0.073

**4-Nitropyridine *N*-oxide in water (mixed excitation)**					
TD-PBE0 (LR)	3.989	3.797(–0.192)	–0.097	–0.095	
TD-PBE0 (cLR)	3.989	3.827(–0.162)	–0.097		–0.065
TD-CAM-B3LYP (LR)	4.196	4.028(–0.168)	–0.065	–0.103	
TD-CAM-B3LYP (cLR)	4.196	4.067(–0.129)	–0.065		–0.064
BSE	3.966	3.687(–0.279)	–0.070	–0.141	–0.068

4-Nitropyridine *N*-oxide is an organic probe used to assess the nature of solvents following a Kamel–Taft type of analysis.^103^ A previous throughout theoretical analysis of the solvatochromism of this probe is available,^104^ and demonstrates that, none of the available LR or SS PCM model is able to describe the solvent effects in a TD-DFT context, as the observed spectral changes come from a fine interplay of several effects. For this compound, the obtained conclusions are similar to the PNA case: both the LR and SS contributions are non-negligible even though the SS dynamic shift is somehow larger. Adding the LR and SS TD-DFT contributions, the overall (*Ω* – *Ω*_0_) shift amounts to –0.16 eV and –0.17 eV with PBE0 and CAM-B3LYP in water, respectively, a shift that can be compared to the –0.21 eV value obtained within BSE. The experimental gas-phase excitation energy obtained through extrapolation in ref. 104 is 3.80 eV, and one notices that the BSE value is reasonably close to that estimate. In benzene, the measurement gives 3.52 eV,^103^ corresponding to a solvatochromic shift of –0.28 eV, a value that BSE can reproduce (–0.27 eV), whereas TD-DFT in unable to do so. In water, the hydrogen bonds with the negatively charged oxygen atom of the probe play a crucial role, and a blueshift is observed (the excitation maximum takes place at 3.94 eV),^103^ an effect that all continuum approaches logically fail to capture.

## 5 Further discussion

**Fig. 3:**
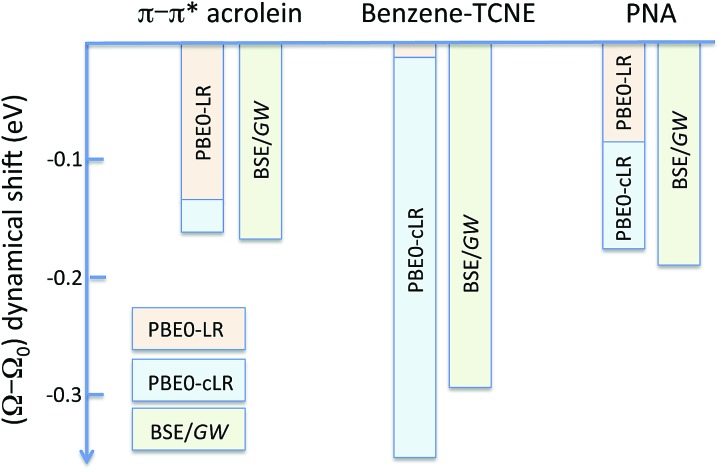
Schematic representation of the (*Ω* – *Ω*_0_) dynamic solvent induced shifts for the π–π* excitation in acrolein, the CT excitation in the benzene–TCNE complex, and the (planar) PNA mixed excitation. By dynamic shift, we mean the effect of switching the fast *v*^reac^(*ε*_∞_) PCM reaction field on top of the (slow) ground-state *v*^reac^(*ε*_0_) PCM response. The represented data correspond to the (*Ω* – *Ω*_0_) shifts given in Tables 1–3.

We summarise in Fig. 3 the principal findings of the present study, namely that the BSE/*GW* formalism accounts for both LR and SS shifts, a fact demonstrated for a large variety of transitions, including excitations where one of the two terms is dominant and excitations for which both are needed. While the partitioning between BSE LR and SS terms is provided in eqn (17b)–(17c), it is interesting to analyse now the contributions to the BSE dynamic shift originating from the solvent-induced renormalisation of the ionisation potential and electronic affinity, namely the variation of the *GW* HOMO–LUMO gap in the solvent, and from the reduction of the electron–hole interaction contained in the *ab*|*W*|*ij* terms.

For the π–π* transition in acrolein, with a shift captured by the LR scheme only at the TD-DFT level, the decomposition of the (*Ω* – *Ω*_0_) energy difference confirms that the variation of the BSE SS-like Δ*ε* – *W* component (eqn (17b)) does not contribute to the shift. Such a result may seem surprising since, as expected, the Δ*ε*^*GW*^ HOMO–LUMO gap becomes smaller by 2.34 eV upon “switching” *v*^reac^(*ε*_∞_), consistent with an (absolute) polarisation energy of *ca.* 1.1–1.2 eV that affects the IP and AE of hydrated compounds compared to the gas phase.^54^ However, very remarkably, the *W* electron–hole binding energy is also decreased by 2.35 eV. Namely, the screening by the fast reaction field reduces similarly the occupied-to-virtual (*ε*_*a*_^*GW*^ – *ε*_*i*_^*GW*^) *GW* gap and the electron–hole binding energy (see Fig. 1). As such, only the *v*^reac^(*ε*_∞_) LR terms (eqn (17c)) explain the solvatochromic effect, consistent with the TD-DFT results.

We now turn to the opposite situation of a CT excitation for which the solvatochromic shift can only be described by SS implementations, such as cLR, within TD-DFT. In the case of the benzene–TCNE complex, the LR *ia*|*v*^reac^(*ε*_∞_)|*bj* contributing matrix elements become very small, as a logical consequence of the small overlap between the donor and acceptor wavefunctions. As such the only contribution to the (*Ω* – *Ω*_0_) shift originates from the variation of the SS-like Δ*ε* – *W* term. The impact of *v*^reac^(*ε*_∞_) leads to the reduction of the Δ*ε* average gap by 1.60 eV, while the electron–hole binding energy *W* reduces by a smaller 1.31 eV amount, accounting for most of the (*Ω* – *Ω*_0_) = –0.29 eV redshift reported in Table 2.

An important consequence of the present analysis, showing that, in BSE/*GW* theory, SS shifts result from the competition between the reduction of the *GW* occupied-to-virtual (*ε*_*a*_^*GW*^ – *ε*_*i*_^*GW*^) energy gaps and the electron–hole *ab*|*W*|*ij* binding energies, is that both electron–electron and electron–hole interactions must be treated on the same footing, namely here through the screened Coulomb potential *W*. In particular, BSE + PCM calculations starting from KS eigenstates generated with exchange-correlation functionals optimally-tuned, so as to generate the correct gas phase HOMO–LUMO gap, might not deliver the correct SS contribution to optical excitation energy shifts.

**Fig. 4:**
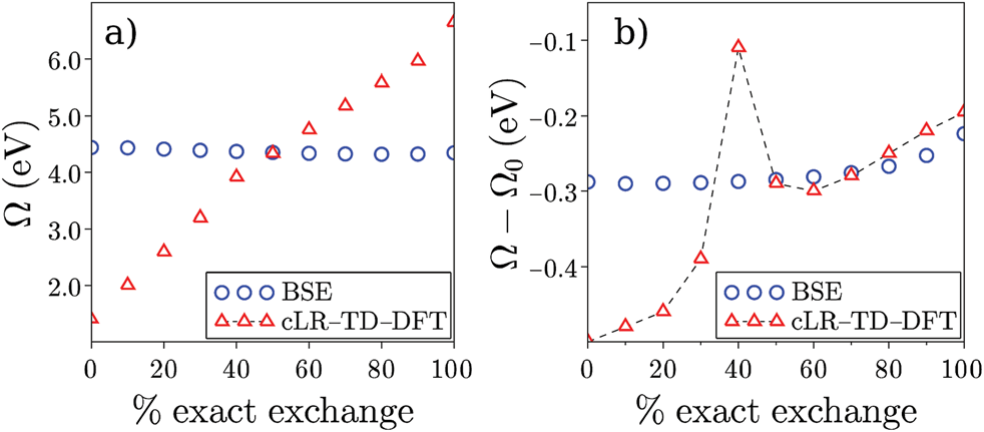
(a) Evolution of the PNA_perp_ excitation energy *Ω* in solution as a function of the percentage of exact exchange *α* in the PBE(*α*) functional. Triangles correspond to the cLR-TD-DFT results while circles indicate BSE/ev*GW*@PBE(*α*) data. (b) Dynamic shift (*Ω* – *Ω*_0_) obtained with cLR-TD-DFT (triangles) and BSE/ev*GW*@PBE(*α*) (circles). The inconsistent value appearing with TD-DFT for *α* = 40% is due to the fact that two excited states of mixed character are nearly degenerate for that EEX ratio. Energies are in eV.

Finally, while Table 2 indicates a large variability of the SS (cLR) TD-DFT (*Ω* – *Ω*_0_) dynamic shift as a function of the chosen XC functional, it is interesting to emphasize the stability of the BSE/ev*GW* data with respect to the input KS eigenstates used to build the ev*GW* electronic energy levels and the screened Coulomb potential *W*. This is illustrated in Fig. 4a where we plot the PNA_perp_ excitation energy in water with the cLR-TD-PBE(*α*) (open triangles) and BSE/ev*GW*@PBE(*α*) (open circles) methods. Here *α* indicates the percentage of exact exchange (EEX) used in the hybrid functional and going from 0% (PBE) to 100% (HFPBE) in Fig. 4. While the TD-DFT excitation energy is shown to increase steeply as a function of *α*, the BSE data are much more stable, with a variation of *ca.* 0.12 eV from (*α* = 0%) to (*α* = 90%). The stability of gas phase BSE/ev*GW* excitation energies with respect to the starting KS starting point has been documented in previous benchmark studies,^47^,^49^ and is therefore shown here to pertain for condensed-phase calculations. In fact, plotting now in Fig. 4b the dynamic (*Ω* – *Ω*_0_) solvatochromic shifts, we observe again a large variability of the TD-PBE(*α*) cLR shifts (the LR contribution is vanishingly small), while again the BSE shifts are extremely stable, evolving from –0.29 eV to –0.25 eV with *α*. To rationalize this result, we recall that BSE calculations are performed on top of partially self-consistent ev*GW* calculations where the corrected electronic energy levels are self-consistently reinjected during the construction of *G* and *W*. This self-consistent treatment of the quasiparticle energies {*ε*_*n*_^*GW*^} and screened Coulomb potential *W* leads to a large stability of the BSE Hamiltonian and reaction field, with a small residual dependency on the starting KS eigenstates due to the fact that the {*φ*_*n*_} eigenstates are unchanged. Such a stability of the BSE/ev*GW* excitation energies and solvent induced shifts, that allows us to alleviate the standard problem of the proper choice of the XC functional central in TD-DFT, is one of the interesting features of the present scheme. As a fair tribute to TD-DFT, we observe however that the cLR shift matches closely that of BSE for a large exact exchange ratio. It is indeed known that large amounts of exact exchange are required in TD-DFT to obtain an accurate description of the ground-to-excited density variation for CT excitations.^17^

## 6 Conclusions

We have demonstrated that the Bethe–Salpeter equation (BSE) formalism combined with the PCM approach straightforwardly includes, on the same footing, both linear-response (LR) and state-specific (SS) contributions to the solvatochromic shifts. This result was established on analytic grounds as well as by comparing the numerical results obtained for several molecules or complexes using BSE + PCM or TD-DFT + PCM approaches, the latter being performed with both the LR and cLR formalisms. To the best of our knowledge, such a consistent theoretical approach simultaneously providing both LR and SS solvent shifts remains unavailable in the TD-DFT world, and has been developed previously, only for theories presenting a less favourable scaling with system size, *e.g.*, ADC(2).^26^

Beyond the standard LR *ab*|*v*^reac^(*ε*_∞_)|*ij* matrix element contributions, where *v*^reac^(*ε*_∞_) is the PCM reaction field to the electronic excitation, the proper inclusion of SS shifts hinges on the incorporation of *v*^reac^(*ε*_∞_) in the screened Coulomb potential *W*. This dressed screened Coulomb potential renormalises both electron–electron and electron–hole interactions, namely both the *GW* quasiparticle energies for occupied/virtual electronic levels and the BSE electron–hole (excitonic) binding energy. Ground-state polarisation effects are accounted for, in the present non-equilibrium scheme, by starting our BSE/*GW* calculations with KS eigenstates generated at the DFT + PCM(*ε*_0_) level.

Following previous studies related to merging the *GW* formalism with discrete polarisable models,^56^–^58^ the same analysis can be straightforwardly applied to BSE calculations combined with other polarisation models.^55^,^57^,^105^ Specifically, the use of an explicit (molecular) description of the environment, combined with, *e.g.*, empirical force fields, should allow us to account for ground-state electrostatic field effects induced by the solvent molecule static multipoles, an important contribution in the case of polar solvents that cannot be captured by the PCM model. As shown in the case of the π–π* transition in aqueous acrolein,^89^,^91^ such contributions can explain the difference between the experimental shift (–0.53 eV) and the ∼–0.25 eV redshifts obtained with the present BSE or TD-DFT-LR formalisms combined with the PCM.

## A From Δ*ε*^*GW*^ to the Born solvation energy model

In this Appendix, we justify eqn (18a). As shown in ref. 54, the variation of the *ε*_*n*_^*GW*^ quasi-particle energies can be simply expressed within the so-called static Coulomb-hole (COH) plus screened-exchange (SEX) approximation to the self-energy *Σ*^*GW*^, namely

25 Δ*Σ*^*GW*^ ≃ Δ*Σ*^SEX^ + Δ*Σ*^COH^,

with the following matrix elements:26
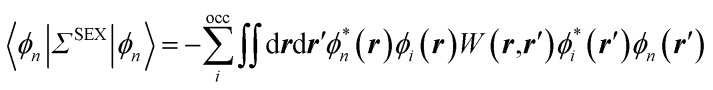

27
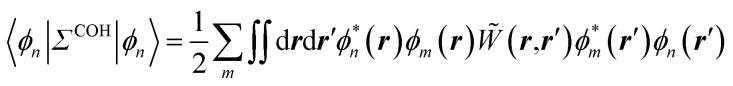
where *W* is taken in the low-frequency optical limit and *W* = *W* – *v*. We therefore get for the variation Δ*ε*_*n*_^*GW*^:28
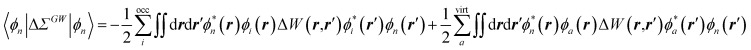
where {*φ*_*n*_} represents the frozen ground-state eigenstates generated at the DFT + PCM(*ε*_0_) level. Here, one can expect the principal contribution to eqn (28) to come from the reaction field induced by the monopolar terms associated with the unit charge density φ*n(***r***)*φ*_*n*_(***r***) = |*φ*_*n*_(***r***)|^2^, *i.e.*,29
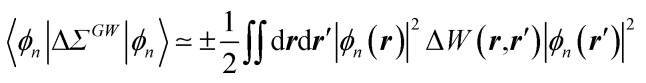
with the + or – sign depending on whether *n* describes an empty (*a*) or an occupied (*i*) state. All other contributions are expected to be smaller, implying most dipole–dipole interactions between neutral codensities since, by orthogonalization, 
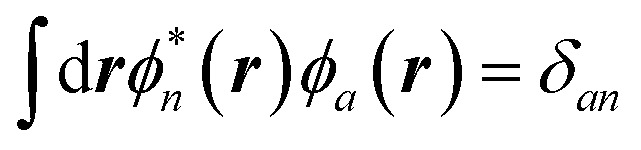
 and 
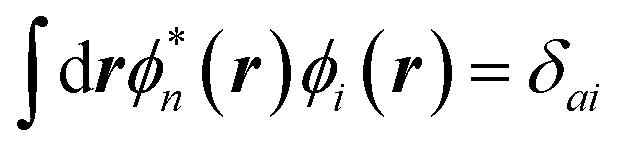
. A similar argument was provided and validated by Neaton and co-workers in their study of the screening of molecular energy levels by metallic electrodes,^106^ in which they assume that Δ*W* is slowly varying over the solute cavity, that is, the monopolar term is dominant.

## B Relation between Δ*W* and *v*^reac^

Working out eqn (14) and (16) to lower order in *v*^reac^ = *vχ*^PCM^*v* leads to:
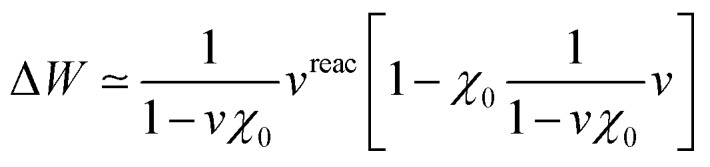
 
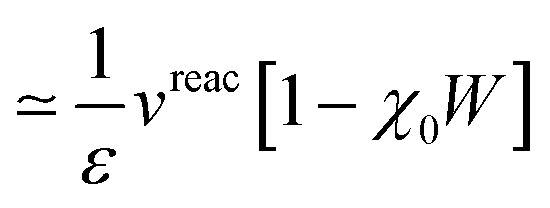
with *χ*_0_, *ε* = 1 – *vχ*_0_, and *W* being the solute free-electron susceptibility, dielectric matrix and screened Coulomb potential in the absence of the PCM. Neglecting the internal response of the QM solute in front of the PCM “reservoir”, namely taking *χ*_0_ → 0, one obtains that indeed Δ*W* is equal to the reaction field.

## Conflicts of interest

There are no conflicts to declare.

## Supplementary Material

Supplementary information
