# Supplementary material for: The Bethe–Salpeter formalism with polarisable continuum embedding: reconciling linear-response and state-specific features
Source: Chem Sci. 2018 Apr 5;9(19):4430–43. doi: 10.1039/c8sc00529j (PMC5956976; doi:10.1039/c8sc00529j)
Supplement: Supplementary file 1 [file SC-009-C8SC00529J-s001.pdf]

Electronic Supplementary Material (ESI) for Chemical Science.
This journal is © The Royal Society of Chemistry 2018

Journal Name

ARTICLE TYPE

Cite this: DOI: 10.1039/xxxxxxxxxx

# The Bethe-Salpeter Formalism with Polarisable Continuum Embedding: Reconciling Linear-Response and State-Specific Features: ESI

Ivan Duchemin,*[a] Ciro A. Guido,[b,c] Denis Jacquemin,[b] and Xavier Blase*[a]

Received Date
Accepted Date

DOI: 10.1039/xxxxxxxxxx

www.rsc.org/journalname

[a] INAC, SP2M/L_Sim, CEA/UJF Cedex 09, 38054 Grenoble, France; E-mail: ivan.duchemin@cea.fr
[b] Laboratoire CEISAM - UMR CNR 6230, Université de Nantes, 2 Rue de la Houssinière, BP 92208, 44322 Nantes Cedex 3, France.
[c] Laboratoire MOLTECH - UMR CNRS 6200, Université de Angers, 2 Bd Lavoisier, 49045 Angers Cedex, France.
[d] Grenoble Alpes University, CNRS, Institut Néel, F-38042 Grenoble, France. E-mail: xavier.blase@neel.cnrs.fr

## S1 Cartesian coordinates

### S1.1 Acrolein

| | | | |
|---|---|---|---|
| 6 | −0.150509 | −0.742320 | −0.000000 |
| 8 | −1.221242 | −1.319646 | −0.000000 |
| 1 | 0.802823 | −1.302877 | 0.000000 |
| 6 | −0.000000 | 0.724345 | 0.000000 |
| 6 | 1.218487 | 1.276311 | 0.000000 |
| 1 | −0.905397 | 1.314305 | −0.000000 |
| 1 | 1.363815 | 2.344937 | 0.000000 |
| 1 | 2.100824 | 0.650783 | 0.000000 |

### S1.2 Indigo

| | | | |
|---|---|---|---|
| 6 | −0.588979 | 2.930950 | 0.000000 |
| 6 | 0.813957 | 2.793967 | 0.000000 |
| 6 | 1.639213 | 3.915686 | 0.000000 |
| 6 | 1.027288 | 5.169382 | 0.000000 |
| 6 | −0.369194 | 5.310246 | 0.000000 |
| 6 | −1.190632 | 4.186090 | 0.000000 |
| 6 | −1.161448 | 1.574198 | 0.000000 |
| 6 | 0.022774 | 0.682297 | 0.000000 |
| 1 | 2.716133 | 3.822424 | 0.000000 |
| 1 | 1.648545 | 6.053968 | 0.000000 |
| 1 | −0.803712 | 6.298973 | 0.000000 |
| 1 | −2.268631 | 4.267593 | 0.000000 |
| 1 | 2.083732 | 1.042213 | 0.000000 |
| 6 | −0.022774 | −0.682297 | 0.000000 |
| 6 | 1.161448 | −1.574198 | 0.000000 |
| 6 | 0.588979 | −2.930950 | 0.000000 |
| 6 | −0.813957 | −2.793967 | 0.000000 |
| 1 | −2.083732 | −1.042213 | 0.000000 |
| 6 | 1.190632 | −4.186090 | 0.000000 |
| 6 | −1.639213 | −3.915686 | 0.000000 |
| 6 | 0.369194 | −5.310246 | 0.000000 |
| 1 | 2.268631 | −4.267593 | 0.000000 |
| 6 | −1.027288 | −5.169382 | 0.000000 |
| 1 | −2.716133 | −3.822424 | 0.000000 |
| 1 | 0.803712 | −6.298973 | 0.000000 |
| 1 | −1.648545 | −6.053968 | 0.000000 |
| 8 | −2.335875 | 1.199345 | 0.000000 |
| 8 | 2.335875 | −1.199345 | 0.000000 |
| 7 | 1.161448 | 1.448720 | 0.000000 |
| 7 | −1.161448 | −1.448720 | 0.000000 |

### S1.3 *para*nitroaniline

| | | | |
|---|---|---|---|
| 6 | 0.003256 | 1.362299 | 1.206029 |
| 6 | 0.003256 | 1.362299 | −1.206029 |
| 6 | 0.003256 | −0.026210 | −1.212800 |
| 6 | 0.003326 | −0.704029 | −0.000000 |
| 6 | 0.003256 | −0.026210 | 1.212800 |
| 1 | 0.007978 | 1.900133 | −2.144629 |
| 1 | 0.002552 | −0.585344 | −2.134454 |
| 1 | 0.002552 | −0.585344 | 2.134454 |
| 1 | 0.007978 | 1.900133 | 2.144629 |
| 7 | 0.000852 | −2.167773 | −0.000000 |
| 8 | −0.000237 | −2.739317 | 1.093615 |
| 8 | −0.000237 | −2.739317 | −1.093615 |
| 7 | 0.070018 | 3.470230 | 0.000000 |
| 1 | −0.302755 | 3.901738 | −0.829058 |
| 1 | −0.302755 | 3.901738 | 0.829058 |
| 6 | −0.000990 | 2.078321 | 0.000000 |

### S1.4 *para*nitroaniline (perp)

| | | | |
|---|---|---|---|
| 6 | 0.000000 | 1.205238 | 1.357916 |
| 6 | −0.000000 | −1.205238 | 1.357916 |
| 6 | −0.000000 | −1.217403 | −0.034182 |
| 6 | 0.000000 | −0.000000 | −0.703823 |
| 6 | 0.000000 | 1.217403 | −0.034182 |
| 1 | −0.000000 | −2.131519 | 1.913615 |
| 1 | −0.000000 | −2.135586 | −0.598912 |
| 1 | 0.000000 | 2.135586 | −0.598912 |
| 1 | 0.000000 | 2.131519 | 1.913615 |
| 7 | 0.000000 | −0.000000 | −2.174543 |
| 8 | 0.000000 | 1.093909 | −2.743861 |
| 8 | −0.000000 | −1.093909 | −2.743861 |
| 7 | 0.000000 | 0.000000 | 3.485857 |
| 1 | −0.855647 | 0.000000 | 3.997970 |
| 1 | 0.855647 | −0.000000 | 3.997970 |
| 6 | 0.000000 | 0.000000 | 2.072561 |

### S1.5 benzene-TCNE (from Ref. 1)

### S1.6 4-nitropyridine *N*-oxide

| | | | |
|---|---|---|---|
| 6 | 0.000000 | 1.188108 | 1.401430 |
| 6 | −0.000000 | −1.188108 | 1.401430 |
| 6 | 0.000000 | −1.205316 | 0.027020 |
| 6 | 0.000000 | −0.000000 | −0.668649 |
| 6 | 0.000000 | 1.205316 | 0.027020 |
| 1 | 0.000000 | −2.062566 | 2.029800 |
| 1 | 0.000000 | −2.142516 | −0.505527 |
| 1 | 0.000000 | 2.142516 | −0.505527 |
| 1 | 0.000000 | 2.062566 | 2.029800 |
| 7 | 0.000000 | −0.000000 | −2.123036 |
| 8 | 0.000000 | 1.097128 | −2.690242 |
| 8 | −0.000000 | −1.097128 | −2.690242 |
| 7 | −0.000000 | 0.000000 | 2.111226 |
| 8 | −0.000000 | 0.000000 | 3.368562 |


This journal is © The Royal Society of Chemistry [year]

## References

1 T. Stein, L. Kronik and R. Baer, *J. Am. Chem. Soc.*, 2009, **131**, 2818–2820.

This journal is © The Royal Society of Chemistry [year]
